# The Effect of Speech Repetition Rate on Neural Activation in Healthy Adults: Implications for Treatment of Aphasia and Other Fluency Disorders

**DOI:** 10.3389/fnhum.2018.00069

**Published:** 2018-02-27

**Authors:** Sarah Marchina, Andrea Norton, Sandeep Kumar, Gottfried Schlaug

**Affiliations:** ^1^Music, Stroke Recovery, and Neuroimaging Laboratories, Department of Neurology, Harvard Medical School, Harvard University, Boston, MA, United States; ^2^Beth Israel Deaconess Medical Center, Boston, MA, United States

**Keywords:** speech rate, overt repetition, fMRI, bilateral activation, temporal lobes, right-hemisphere language networks, fluency, speech-motor function

## Abstract

Functional imaging studies have provided insight into the effect of rate on production of syllables, pseudowords, and naturalistic speech, but the influence of rate on repetition of commonly-used words/phrases suitable for therapeutic use merits closer examination.

**Aim:** To identify speech-motor regions responsive to rate and test the hypothesis that those regions would provide greater support as rates increase, we used an overt speech repetition task and functional magnetic resonance imaging (fMRI) to capture rate-modulated activation within speech-motor regions and determine whether modulations occur linearly and/or show hemispheric preference.

**Methods:** Twelve healthy, right-handed adults participated in an fMRI task requiring overt repetition of commonly-used words/phrases at rates of 1, 2, and 3 syllables/second (syll./sec.).

**Results:** Across all rates, bilateral activation was found both in ventral portions of primary sensorimotor cortex and middle and superior temporal regions. A repeated measures analysis of variance with pairwise comparisons revealed an overall difference between rates in temporal lobe regions of interest (ROIs) bilaterally (*p* < 0.001); all six comparisons reached significance (*p* < 0.05). Five of the six were highly significant (*p* < 0.008), while the left-hemisphere 2- vs. 3-syll./sec. comparison, though still significant, was less robust (*p* = 0.037). Temporal ROI mean beta-values increased linearly across the three rates bilaterally. Significant rate effects observed in the temporal lobes were slightly more pronounced in the right-hemisphere. No significant overall rate differences were seen in sensorimotor ROIs, nor was there a clear hemispheric effect.

**Conclusion:** Linear effects in superior temporal ROIs suggest that sensory feedback corresponds directly to task demands. The lesser degree of significance in left-hemisphere activation at the faster, closer-to-normal rate may represent an increase in neural efficiency (and therefore, decreased demand) when the task so closely approximates a highly-practiced function. The presence of significant bilateral activation during overt repetition of words/phrases at all three rates suggests that repetition-based speech production may draw support from either or both hemispheres. This bihemispheric redundancy in regions associated with speech-motor control and their sensitivity to changes in rate may play an important role in interventions for nonfluent aphasia and other fluency disorders, particularly when right-hemisphere structures are the sole remaining pathway for production of meaningful speech.

## Introduction

In healthy individuals, fluent speech production involves a series of integrated commands [e.g., *predictive commands* to the motor cortex to establish a target (feedforward); *assessment/analysis* commands to auditory-motor regions to evaluate accuracy of output compared to the predicted target (feedback); and when a mismatch is detected, *corrective commands* to motor cortices to both initiate and respond to those auditory targets in the feedforward/feedback loop (modification/correction)] (Houde and Jordan, 1998; Tourville et al., 2008; Houde and Nagarajan, 2011). In contrast, individuals with damage to speech-motor regions or their associated network connections [as in the case of stroke or traumatic brain injury (TBI)] are left with disruptions to speech-motor control that result in fluency disorders (Kent, 2000; van Lieshout et al., 2007; Marchina et al., 2011; Wang et al., 2013; Pani et al., 2016) that can slow, impair, or prevent production of meaningful speech.

Syllable production rate has been recognized as a sensitive clinical indicator for detecting and diagnosing speech-motor disorders [e.g., dysarthria/anarthria (Kent et al., 1987), apraxia of speech (Kent and Rosenbek, 1983; Ziegler, 2002; Aichert and Ziegler, 2004; Ogar et al., 2006), and stuttering (Logan and Conture, 1995; Arcuri et al., 2009)], and syllables per minute (spm) has long been considered a precise, yet flexible measure of speech rate/fluency capable of evaluating both the severity of disordered speech in impaired populations and the efficiency (in terms or rate and fluency) of communication in healthy speakers (Cotton, 1936; Kelly and Steer, 1949; Grosjean and Deschamps, 1975; Costa et al., 2016).

Neuroimaging studies using a variety of syllable rates have reported modulation of activity within speech-motor networks (Wildgruber et al., 2001; Riecker et al., 2005, 2006) as well as modulation of activation in speech-motor regions bilaterally in response to changes in syllable-production rate (Wildgruber et al., 2001; Riecker et al., 2005; Liegeois et al., 2016). Others have examined rate effects in the auditory domain using passive listening to words, syllables, and non-speech noise (Binder et al., 1994; Dhankhar et al., 1997; Noesselt et al., 2003), or single syllables and pseudowords (Wildgruber et al., 2001; Riecker et al., 2006, 2008; Liegeois et al., 2016) rather than meaningful words or phrases.

Despite the common assumption that covert and overt speech use the same processes and share neural mechanisms, studies have reached different conclusions. Palmer et al. (2001) showed similar activation patterns for both response modes once motor activity associated with overt speech was removed; others found distinctly different patterns of neural activation for covert and overt speech. Both Huang et al. (2002) and Shuster and Lemieux (2005) observed stronger activation for overt speech than for covert speech; Brumberg et al. (2016) noted that speech output intensity led to better identification of neural correlates for overt, but not covert sentence production; and Basho et al. (2007) suggested that without overtly spoken responses, the scope of language tasks for functional magnetic resonance imaging (fMRI) would be limited by the inability to assess subjects’ participation, obtain behavioral measures, or monitor responses. Overt repetition of real words is particularly important for studies of speech repetition rate because it enables continuous monitoring of task compliance and accuracy of responses/consistency of adherence to rate within and across subjects.

Patients with speech-motor disorders typically respond better to therapeutic interventions that use slower rates (e.g., Southwood, 1987; Yorkston et al., 1999; Duffy and Josephs, 2012). Based on existing evidence for hemispheric specialization from studies that found left-hemisphere dominance for rapid temporal processing and right-hemisphere sensitivity to slow transitions/longer durations (McGettigan and Scott, 2012) and studies of patients with large left-hemisphere lesions (e.g., Schlaug et al., 2009; Marchina et al., 2011; Zipse et al., 2012), we hypothesized that hemispheric response to speech-production rates might differ. To capture potential shifts in activation, we chose three frequency rates- *slow* (1 syll./sec.) to simulate the rate used with nonfluent aphasic patients in the early stages of treatment, *moderate* (2 syll./sec.) to approximate the rate used in therapy as patients improve, and *closer-to-normal* (3 syll./sec.) which, in general, is slower than that of a typical adult speaker but remains within the range for speakers of American English [∼2.1 syll./sec. (Ruspantini et al., 2012); ∼2.83 syll./sec. (Costa et al., 2016); ∼3.67 syll./sec. (Robb et al., 2004); ∼3.75 syll./sec. (Blank et al., 2002); ∼3–6 syll./sec. (Levelt, 2001); ∼4–5 syll./sec. (Alexandrou et al., 2016)].

The incremental range of speech rates used in our study was designed to (1) provide a more comprehensive view of the healthy brain’s regional response to rate changes within the well-described perisylvian network (Binder et al., 1997; Hickok and Poeppel, 2004; Catani et al., 2005; Crosson et al., 2007; Oliveira et al., 2017), (2) identify regions capable of supporting recovery from fluency disorders characterized by impaired initiation and/or slow, halting speech production, (3) gain important insights for the development of treatment protocols that can be adapted as fluency improves over the course of treatment/recovery, and (4) examine neural response to changes in rate in terms of linear and/or non-linear effects and hemispheric laterality.

## Materials and Methods

### Participants

Twelve healthy, right-handed native speakers of American English (five females, seven males) ranging in age from 30 to 69 years (mean age: 52.0 ± SD: 10.1 years), with no history of neurological, speech, language, or hearing disorders were recruited. The protocol was approved by Beth Israel Deaconess Medical Center’s Institutional Review Board, and all subjects gave written informed consent in accordance with the Declaration of Helsinki.

### Behavioral Testing

Handedness was assessed by self-report using measures adapted from The Edinburgh Inventory (Oldfield, 1971). To ensure that cognition fell within a normal range, subjects completed the Shipley/Hartford Institute of Living assessment (Shipley, 1940), which correlates highly with the Wechsler Adult Intelligence Scale full scale IQ (Paulson and Lin, 1970). Subjects’ IQ equivalents (derived from Shipley scores) all fell within normal limits (mean: 122.09 ± SD: 11.53) and thus, all were included in the data analyses.

### Experimental Stimuli

**FIGURE 1:**
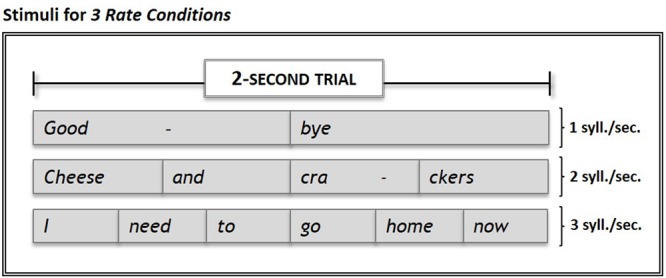
Examples of stimuli used in the experiment. Stimuli set consisted of 15 commonly-used words and phrases presented in 2-sec. trials at rates of 1, 2, and 3 syll./sec. Subjects’ overt repetitions of stimuli in each of the three *Rate Conditions* were compared to their response during *Silence* (control condition) trials. Within each run, the three rate conditions and the *silence* (control) condition were each sampled five times.

A set of 15 stimuli consisting of commonly-used 2-, 4-, and 6-syllable words/phrases (e.g., “*Goodbye”*; “*Cheese and crackers”*; “*I need to go home now”*) was recorded at rates of 1, 2, or 3 syll./sec. respectively, by a trained, native speaker of American English using Adobe Audition 1.5 software (Adobe, San Jose, CA, United States). The total time for each stimulus equaled 2 s. For each rate, all syllables were produced with equal duration (see **Figure 1**).

### Task Design

We used an overt repetition task in order to monitor subjects’ task compliance, adherence to protocol timing, and repetition rate in each condition, as well as to verify accuracy of repetition rates within and across subjects. Our stimuli consisted of familiar words and commonly-used phrases that hold potential for use in treatment of fluency disorders.

The fMRI protocol was comprised of 6 runs, each with 20 active trials + 2 dummy acquisitions (15 sec./trial; 5 min 30 sec./run). Within each run, experimental conditions (three speech repetition rates: 1, 2, and 3 syll./sec.) were each sampled five times (15 trials), with five control condition (*silence*) trials interspersed. Conditions were pseudo-randomized {i.e., the order of the 20 active trials [5 experimental (*overt repetition*) trials × 3 rates + 5 control (*silence*) trials] was randomized once, and that same order of condition was then used for all runs}. Order of stimuli was randomized independently for each run. For trials presented in the repetition-rate conditions, each stimulus was followed by a short “ding” that served as an auditory cue to begin overt repetition of the target word/phrase. Subjects were instructed to repeat each target exactly as they had heard it, immediately after the cue. For the control (*silence*) trials, no spoken stimuli were presented. Subjects were asked to remain quiet until they heard the auditory cue (*ding*), then take a quick breath and exhale to simulate their preparation for initiation of spoken responses in the experimental (*repetition-rate*) conditions.

Prior to the fMRI experiment, a member of the research team explained the experimental design and what would take place during the scanning session. Subjects were given approximately 20 min to familiarize themselves with the stimuli and practice the tasks and timing with one of the researchers.

During the fMRI experiment, auditory stimuli were presented via MR-compatible, noise-canceling headphones while the subjects lay supine in the scanner. Subjects were asked to hold as still as possible and keep their eyes closed throughout the scanning session to ensure that acquisitions would capture only task-related activation. Subjects’ responses were noted by researchers to verify task compliance.

### Image Acquisition

Functional MRI was performed on a 3T GE whole-body scanner. A gradient-echo EPI-sequence (TR 15 s, TE 25 ms, acquisition time 1.75 s) with a matrix of 64 × 64 was used for functional imaging. 28 contiguous axial slices covering the whole brain resulted in a voxel size of 3.75 mm × 3.75 mm × 5 mm. Image acquisition was synchronized with stimulus onset using Presentation software (Neurobehavioral Systems, Albany, CA, United States). The total scan time including the acquisition of a high-resolution MPRAGE anatomical sequence (voxel resolution of 0.93 mm × 0.93 mm × 1.5 mm) was, on average, 40 min per subject.

We used a jittered, sparse temporal sampling design with precisely-timed acquisitions to capture task-related activation and reduce/eliminate auditory artifacts associated with stimulus presentation, auditory cueing, and scanner noise. The *Silence* condition was designed to control for activation associated with the preparatory breath and initiation of the speech-motor response necessary for overt repetition in the rate conditions. Although the TR remained constant at 15 s, the delay between subjects’ responses and onset of MR acquisition was varied by moving the task block within the 15 s time frame. These shifts yielded stacks of axial images with delays of 3.5, 4.5, 5.5, and 6.5 s after the auditory cue. By combining the data from the four jitter points, we were able to capture peak hemodynamic response for each condition while allowing for individual timing differences between subjects and brain regions. Ten of the 12 subjects completed all six functional runs. Due to unforeseen scanner time constraints, the sessions of the two remaining subjects were truncated, and thus, they completed only four and three runs, respectively. Nevertheless, all runs of all subjects were included in the analyses.

### fMRI Data Analysis

Data were analyzed using SPM5 (Institute of Neurology, London, United Kingdom) implemented in Matlab (Mathworks, Natick, MA, United States). Pre-processing included realignment and unwarping, spatial normalization, and spatial smoothing using an isotropic Gaussian kernel (8 mm). Condition and subject effects were estimated using a general linear model (Friston, 2002). The effect of global differences in scan intensity was removed by scaling each scan in proportion to its global intensity. Low-frequency drifts were removed using a temporal high-pass filter with a cutoff of 128 s (default setting).

As is the case with sparse temporal sampling design, there was no temporal auto correlation between the images. Therefore, we did not convolve our data with the hemodynamic response function, but instead, used the flexible finite impulse response, which averages the BOLD response at each post-stimulus time point. The data were analyzed on a single subject basis in order to enter the individual contrasts into a random effects analysis. One-sample *t-*tests that included a ventricular mask were calculated individually for each syllable rate by applying a significance threshold of *p* < 0.01 and correcting for multiple comparisons using the false discovery rate (FDR). For an analysis of variance (ANOVA) with three levels, we used the full factorial design and corrected for family wise error (FWE) at a significance level of *p* < 0.05.

Local maxima in each cluster of the conjunction analysis were extracted to create a spherical ROI (10 mm). The ROIs were overlaid on each subject’s contrast images for 1-, 2-, and 3-syllable rates > silence; mean beta-values were extracted for each ROI, then used for the repeated measures ANOVA in SPSS.

## Results

### Speech Repetition Rates: 1, 2, and 3 syll./sec. vs. Silence (Control Condition)

**FIGURE 2:**
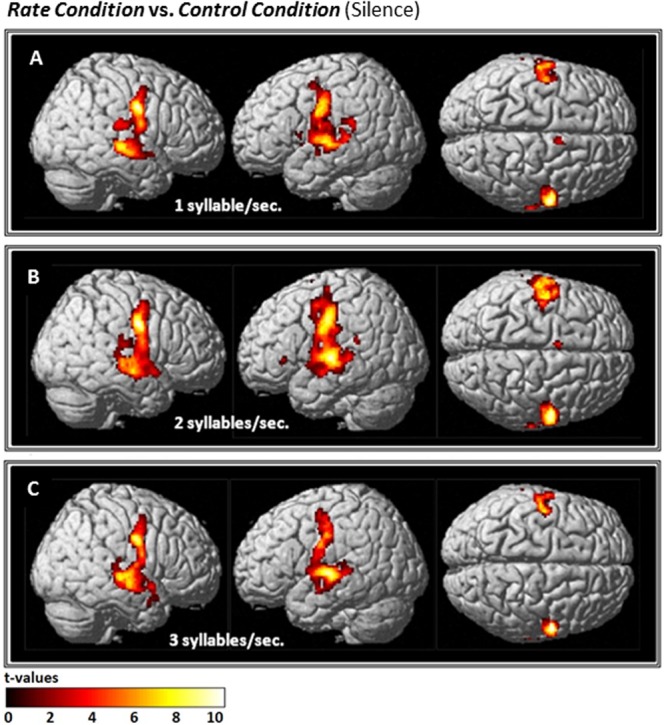
Effects of the three individual *Rate Conditions* vs. *Silence* (control condition) **(A–C)**: *t*-Tests comparing 1-, 2-, and 3-syll./sec. *Rates* vs. *Silence* revealed bilateral patterns of activation in a group of 12 healthy subjects during overt repetition of words and phrases spoken at three different rates. Highly-significant linear increases were observed at all three rates in the right-hemisphere and at the 1- and 2-syll./sec. rates in the left. Left-hemisphere activation at the *closer-to-normal* speech rate (3 syll./sec.), though still significant, was less robust. Statistical maps are FDR 0.01 corrected; the extent threshold is 20 voxels.

Compared with *Silence* (control condition), all *Rate* conditions yielded extensive clusters of activation in bilateral speech-motor regions that included primary motor and adjacent premotor cortices, superior temporal gyri (STG), superior temporal sulci (STS), and middle temporal gyri (MTG). In the 1 syll./sec. contrast, additional activation was found in the right cingulate gyrus, and the left insula (see **Figure 2A** and **Table 1A**).

**Table 1A:** One-sample *t*-test: 1 syll./sec. > *silence*, FDR 0.01, voxel threshold 20.

Region	Hemisphere	Extent (voxel)	*t-*value	MNI coordinates		
				*x*	*y*	*z*
Superior temporal gyrus	L	2114	10.96	–62	–34	6
Superior temporal gyrus	L		10.92	–56	–6	4
Superior temporal gyrus	L		9.09	–54	–20	0
Precentral gyrus	R	2170	10.57	60	–6	34
Middle temporal gyrus	R		9.89	58	–8	–10
Precentral gyrus	R		9.50	60	–4	14
Supplementary motor area	R	49	7.55	8	2	66
Precentral gyrus	L	39	7.43	–42	10	10
Insula	L		7.36	–38	6	0
Superior temporal gyrus	L	98	7.01	–50	–42	16
Insula	L		6.41	–56	–32	20
Cingulate gyrus	R	44	6.99	16	20	38
Cingulate gyrus	R		5.95	16	12	40

Similarly, the 2-syll./sec. rate elicited additional activation in the left inferior frontal gyrus (IFG) and left supplementary motor area (SMA) (see **Figure 2B** and **Table 1B**).

**Table 1B:** One-sample *t*-test: 2 syll./sec. > *silence*, FDR 0.01, voxel threshold 20.

Region	Hemisphere	Extent (voxel)	*t-*value	MNI coordinates		
				*x*	*y*	*z*
Middle temporal gyrus	R	2305	12.55	58	–10	–10
Precentral gyrus	R		12.33	60	–4	12
Precentral gyrus	R		10.53	56	–6	32
Middle temporal gyrus	L	3404	11.56	–62	–34	4
Superior temporal gyrus	L		10.40	–56	–8	6
Superior temporal gyrus	L		9.87	–56	–18	–2
Inferior frontal gyrus	L	38	10.28	–36	30	2
Supplementary motor area	L	37	6.79	–6	4	64

For the 3-syll./sec. rate, additional activation was located in the right insula and the left parietal operculum (see **Figure 2C** and **Table 1C**).

**Table 1C:** One-sample *t*-test: 3 syll./sec. > *silence*, FDR 0.01, voxel threshold 20.

Region	Hemisphere	Extent (voxel)	*t-*value	MNI coordinates		
				*x*	*y*	*z*
Precentral gyrus	L	1827	10.23	–60	–4	16
Superior temporal gyrus	L		9.45	–58	–18	0
Superior temporal gyrus	L		9.13	–52	–24	0
Insula	R	1978	9.83	46	–18	4
Middle temporal gyrus	R		9.19	64	–20	–6
Middle temporal gyrus	R		9.12	62	–12	–8
Parietal operculum	L	43	6.36	–38	–34	24

### One-Way ANOVA with Three Levels

**FIGURE 3:**
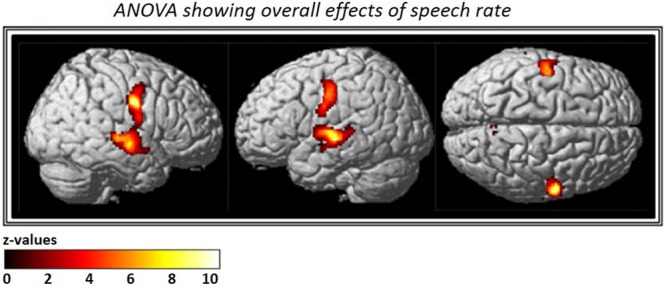
Analysis of variance (ANOVA) showing overall effects of speech repetition rate. Contrast images from the first level analysis for all three speech repetition rates were entered into a full factorial design in order to calculate an ANOVA with three levels. The resulting F-contrast was FWE corrected at a significance level of *p* < 0.05; an extent threshold of 20 voxels was applied.

The ANOVA that included all three *speech rates* vs. *silence* revealed bilateral activation in speech-motor control regions that included the pre- and post-central gyri, superior- and middle- temporal gyri, as well as the STS. In addition, a smaller cluster was found in the right precuneus (see **Figure 3** and **Table 2**).

**Table 2:** Analysis of variance (ANOVA) – FWE, voxel threshold 20.

Region	Hemisphere	Extent (voxel)	*Z*-value	*F-*value	MNI coordinates		
					*x*	*y*	*z*
Superior and middle temporal gyri	R	1392	7.33	62.19	62	–10	–6
Pre- and post-central gyri	R		6.95	50.93	56	–6	32
Pre- and post-central gyri	R		6.16	33.74	56	–4	44
Middle temporal gyrus	L	787	7.58	71.22	–64	–18	–4
Superior temporal gyrus	L		7.10	55.01	–58	–12	4
Middle temporal gyrus	L		5.91	29.73	–62	–34	4
Post-central gyrus	L	611	6.15	33.63	–48	–14	32
Precentral gyrus	L		5.97	30.64	–58	–6	24
Precentral gyrus	L		5.95	30.33	–56	–14	40
Precuneus	R	36	5.33	22.08	2	–60	48

### ROI Analysis

To further explore the pattern seen in the contrast estimates, significant clusters in the conjunction analysis served as the basis for a region of interest (ROI) analysis. Thus, we created two ROIs in each hemisphere using the local maxima in the superior temporal [right: 62 -12 -6; left: -64 -18 -4 (in MNI space)] and middle to inferior motor cortices (left: -48 -14 32; right: 58 -6 32). We then conducted a one-way, repeated measures ANOVA with Bonferroni *post hoc* pairwise comparisons, which revealed a significant difference in mean beta-values within the temporal ROIs bilaterally across syllable rates (right: *F* = 29.36, *p* < 0.001; left: *F* = 30.083, *p* < 0.001).

**FIGURE 4:**
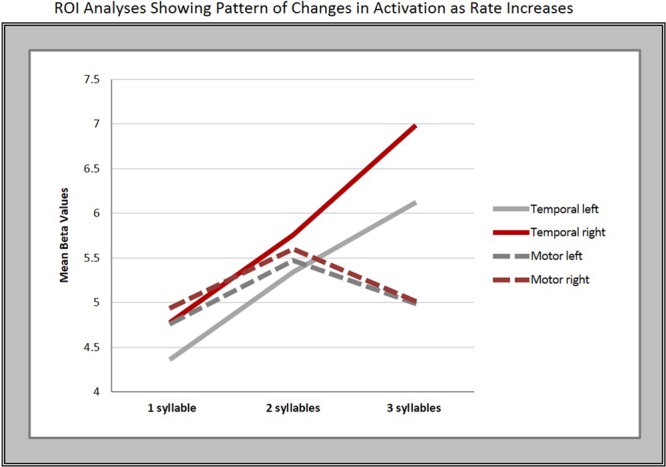
Regions of interest (ROI) analyses. The graphs show the mean beta-values in response to changes in syllable rate in motor and temporal regions bilaterally.

Five of the six pairwise comparisons between the three rates in the temporal ROIs were highly significant (*p* < 0.008), while the 2- vs. 3-syll./sec. contrast in the left hemisphere, though still significant, was less robust at *p* = 0.037 (see **Figure 4** and **Table 3**).

**Table 3:** Pairwise comparisons in the temporal lobes.

Region of interest	Syllable rate		Mean difference	*SE*	Significance (Bonferroni)	95% confidence interval	
	I	II	(I–II)			Lower bound	Upper bound
Left temporal	1	2	–0.967	0.178	*0.001*	–1.469	–0.465
	1	3	–1.734	0.230	*0.000*	–2.382	–1.085
	2	3	–0.767	0.257	*0.037*	–1.491	–0.043
Right temporal	1	2	–0.963	0.243	*0.007*	–1.647	–0.279
	1	3	–2.171	0.295	*0.000*	–3.001	–1.340
	2	3	–1.207	0.310	*0.007*	–2.082	–0.333

*Italics were used to indicate statistical significance.*

In contrast, no significant overall differences in mean beta-values for the different syllable rates were found in the motor cortex ROIs on either the right (*F* = 2.42, *p* = 0.14, Greenhouse–Geisser corrected) or left hemisphere (*F* = 3.003, *p* = 0.07).

## Discussion

The aims of the present study were to (1) examine healthy adults’ neural response to changes in rate during overt repetition of meaningful words/phrases, (2) determine whether such changes are capable of modulating activation within speech-motor regions, and if so, (3) whether those modulations occur in a linear manner and/or show hemispheric preference.

Early lesion studies found language functions to be localized predominantly in the left hemisphere (Broca, 1865; Wernicke, 1874; Geschwind, 1970), but were limited in their ability to link speech function to structure *in vivo*. With the evolution of functional imaging, investigations of both healthy and lesioned brains have provided substantial evidence for bilateral organization of speech production (e.g., Hickok et al., 2000; Hickok and Poeppel, 2004, 2007; Saur et al., 2008). In healthy subjects, speaking rate and/or speech-repetition rate has been studied primarily as a means for understanding speech-motor control. Two recent studies of spontaneous connected speech have led to a greater understanding of the role that speech production networks and cortical regions associated with perception and production play in natural speech. Silbert et al. (2014) used a novel fMRI technique to examine unconstrained, 15-min long, real-life speech narratives and found symmetric bilateral activation in sensorimotor and temporal brain regions. Alexandrou et al. (2017) used MEG to study the perception and production of natural speech at three different rates and not only noted distinct patterns of modulation in cortical regions bilaterally, but highlighted the role of the right temporo-parietal junction in task modulation.

Other fMRI studies have also shown that repetition of simple phrases and/or individual syllables activates bilateral networks (Bohland and Guenther, 2006; Ozdemir et al., 2006; Rauschecker et al., 2008). Despite the fact that the present study’s experimental task involved repetition of stimuli at increasing rates and lengths rather than 2-syllable phrases repeated at a constant rate, overall, the results align with those of Ozdemir et al. (2006) showing bilateral activation in the IFG for motor planning and auditory-motor mapping, primary sensorimotor cortex activation for articulatory action, and the middle- and posterior STG/STS for sensory feedback.

Of particular interest in terms of repetition and rate, Wise et al. (1999) employed a listening/repetition task involving 2-syllable nouns produced at multiple slower rates [10, 20, 30, 40, and 50 words/min (i.e., ranging from 0.33 to 1.67 syll./sec.)], and found bilateral activation associated with word repetition in primary sensorimotor cortices, additional activity in the left anterior insula, posterior pallidum, anterior cingulate gyrus, dorsal brainstem, and rostral right paravermal cerebellum. Increased temporal lobe activation corresponded with rate increases for both listening and repetition conditions, and a linear increase associated with increased repetition rate was seen in the sensorimotor cortex. Some of their findings were confirmed by our data which revealed that activation in the superior temporal cortex increased linearly across all three rates on both hemispheres, while increases in the primary sensorimotor cortices showed no linear rate effects. The increased neural activity observed as speech repetition rate increases lends support to the notion that speech-motor regions modulate in response to task demands (Price et al., 1992; Paus et al., 1996; Sörös et al., 2006; Dunst et al., 2014; Alexandrou et al., 2017). The less robust effect in the left hemisphere seen at faster rates may be due to the fact that speech produced at a closer-to-normal pace is a highly practiced function and therefore, requires no additional regional support (Dunst et al., 2014; Nussbaumer et al., 2015).

Wildgruber et al. (2001) used fMRI and repetition of a simple syllable (/ta/) performed at three different rates (2.5, 4.0, and 5.5 Hz) to determine the independent contributions of cerebral structures that support speech-motor control. Bilateral motor cortices showed a positive correlation with production frequencies. Activation in the right superior temporal lobe increased from 2.5 to 4 Hz, but then decreased. These results differed from those of our study which showed a significant difference and increase across all three rates in the temporal lobe, but not in the motor cortex. The underlying cause of this discrepancy is difficult to discern due to the covert nature of a task involving imagined silent repetition of a single syllable. Moreover, in contrast to overt repetition of commonly-used words/phrases, a nonfluent syllable such as /ta/, is one that would likely deter rather than enhance fluency, and thus, may have become more difficult to “produce” as rates increased. Although Wildgruber and others have argued that speech-motor control can be successfully assessed by covert tasks (Wildgruber et al., 1996; Ackermann et al., 1998; Riecker et al., 2000), when Shuster and Lemieux (2005) used both overt and covert stimuli in a word production task, they concluded that, despite similarities, the BOLD response was not the same for the two modalities.

Furthermore, because our repetition rates (1, 2, and 3 syll./sec.) were somewhat slower than the 2.5, 4.0, and 5.5 Hz used by Wildgruber et al. (2001), they may have engaged the right temporal cortex which has been shown to be particularly sensitive to slow temporal features, and therefore, may underlie the encoding of syllable patterns in speech (Boemio et al., 2005; Abrams et al., 2008). Riecker et al. (2006) also investigated speech-motor control using simple, overt repetition of the syllable /pa/ at six different frequencies (2.0 to 6.0 Hz). There, the rate-to-response functions of the BOLD signal revealed a negative relationship between syllable frequency and the striatum, whereas cortical areas and the cerebellum showed the opposite pattern. Surprisingly, they found no activation in the superior temporal cortex as we did in our study. This is, however, in alignment with Wildgruber et al. (2001) who suggest that fewer resources are required from temporal lobe regions for simple syllable repetition. It may also indicate that a more classical perisylvian network is engaged for speaking meaningful phrases while repetition of a single syllable at a faster pace requires greater support from classical motor-control networks.

Noesselt et al.’s (2003) examination of rate effects used an auditory word presentation task that showed a strong linear correlation between presentation rate and bilateral hemodynamic response in the auditory cortices of the STG. They concluded that because “word presentation rate” modulated activation in these areas, it works in a stimulus-dependent fashion. A similar result was found by Dhankhar et al. (1997) who presented auditory stimuli at rates ranging from 0 to 130 words/min (i.e., 0 to 2.17 words/sec.). They found that the total volume of activation in the STG’s auditory regions increased as the presentation rate increased, peaking at 90 words/min (i.e., 1.5 words/sec.) with a subsequent fall at 130 (i.e., 2.17 words/sec.). In our study, overt speech production at three different rates also elicited linear increases in the STG bilaterally, with a lesser increase in intensity on the left between the 2- and 3-syll./sec. rates. Converging evidence has also identified a potential role for the pSTG in aspects of speech production (Price et al., 1996; Wise et al., 2001; Bohland and Guenther, 2006; Ozdemir et al., 2006). Ozdemir et al. (2006) suggested that activated regions in the temporal cortex may be responsible for providing sensory feedback; Price et al. (1992) found a linear relationship between blood flow and presentation rate of heard words in the right STG. The left STG was activated in response to the words themselves, but not to the rate of presentation.

In this study, the strong superior temporal lobe activation observed in combination with activation in other speech-relevant perisylvian language areas suggests fluid teamwork within the speech-motor circuitry shared by regions that support both motor preparation/execution and sensory feedforward/feedback control for speech production. This is consistent with studies that found evidence for left-hemisphere dominance in rapid temporal processing and right-hemisphere sensitivity to longer durations (McGettigan and Scott, 2012; Han and Dimitrijevic, 2015). Furthermore, clinical studies in patients with large left-hemisphere lesions and nonfluent aphasia (e.g., Schlaug et al., 2008; Zipse et al., 2012), have found right-hemispheric support for repetition of meaningful words at slower rates (1 syll./sec.). Our results confirm that bihemispheric sensorimotor regions, part of the feedforward/feedback control loop for speech production, are actively engaged during paced, overt word/phrase repetition in healthy adults. These findings complement the growing body of evidence provided by lesion studies, and together, advance a more comprehensive picture of the effect of rate on neural activation and its promise for the treatment of nonfluent aphasia and other fluency disorders.

There are, however, a number of limitations/shortcomings which deserve consideration. First, the sample size of 12 is at the smaller end for this kind of studies. Designed as a pilot study to inform future studies with aphasic patients, we elected to proceed with the smallest possible sample that was still large enough to statistically power analyses of our sparse temporal sampling paradigm. Second, we are aware that fMRI is not necessarily the optimal imaging method with regard to temporal resolution for time-sensitive tasks; however, our main objective was to visualize and localize the neural correlates of speech-repetition at different rates. Third, despite recording and assessing the subject’s responses in real time, the study lacked an acoustic measure that could be used to more precisely assess error type/extent and make potential correlations with regions activated during paced repetition, although we think that this error analysis would likely reveal only minor variations of the main findings since the speech repetition rate was modulated on a rather large scale.

## Conclusion

The linear effects seen in superior temporal lobe ROIs suggest that sensory feedback corresponds directly to task demands. The lesser degree of increase in left-hemisphere activation between the 2- and 3-syllable rates may represent an increase in neural efficiency, thus indicating that faster rates are less demanding on regional function in the left hemisphere when the task so closely approximates a highly-practiced function. The overall pattern of bilateral activation during overt repetition, coupled with right-hemisphere dominance in response to changes in speech repetition rate further suggest that interventions aiming to improve speech fluency through repetition could draw support from either or both hemispheres. This bihemispheric redundancy in speech-motor control may play an important role in recovery of speech production/fluency, particularly for patients with large left-hemisphere lesions for whom the right hemisphere is the only option for production of meaningful speech. Results of this investigation may help identify optimal rates for treatment at different stages of recovery, and provide insight for the development of interventions seeking to target nonfluent aphasia and other fluency disorders characterized by impaired initiation and/or slow, halting speech production that are typically treated with repetition-based therapies.

## Author Contributions

Substantial contributions to the conception or design of the work; or the acquisition, analysis, or interpretation of data for the work; drafting the work or revising it critically for important intellectual content; final approval of the version to be published; agreement to be accountable for all aspects of the work in ensuring that questions related to the accuracy or integrity of any part of the work are appropriately investigated and resolved: SM, AN, SK, and GS.

## Conflict of Interest Statement

The authors declare that the research was conducted in the absence of any commercial or financial relationships that could be construed as a potential conflict of interest.
